# Impact of the COVID-19 pandemic on maternal mental health during pregnancy: The CONCEPTION study – Phase I

**DOI:** 10.1192/j.eurpsy.2022.547

**Published:** 2022-09-01

**Authors:** A. Berard, A. Lacasse, Y.-H. Gomez, J. Gorgui, S. Côté, S. King, V. Tchuente, F. Muanda, Y. Lumu, I. Boucoiran, A.-M. Nuyt, C. Quach, E. Ferreira, P. Kaul, B. Winquist, K. O’Donnell, S. Eltonsy, D. Château, J.-P. Zhao, G. Hanley, T. Oberlander, B. Kassai, S. Mainbourg, S. Bernatsky, É. Vinet, A. Brodeur-Doucet, J. Demers, P. Richebé, V. Zaphiratos, C. Wang, X. Wang

**Affiliations:** 1 CHU Sainte-Justine, Research Center, Montreal, Canada; 2 Université du Québec en Abitibi-Témiscamingue, Health Sciences Department, Rouyn-Noranda, Canada; 3 McGill University, Faculty Of Medicine, Montreal, Canada; 4 Western University, Department Of Epidemiology & Biostatistics, London, Canada; 5 Protestant University in Congo, Family Medicine, Kinshasa, Congo, Republic of; 6 University of Alberta, Department Of Medicine, Edmonton, Canada; 7 University of Saskatchewan, College Of Medicine, Saskatoon, Canada; 8 McGill University, Douglas Research Center, Montreal, Canada; 9 University of Manitoba, College Of Pharmacy, winnipeg, Canada; 10 University of Manitoba, Manitoba Center For Health Policy, Winnipeg, Canada; 11 University of British Columbia, Department Of Obstetrics & Gynaecology, Vancouver, Canada; 12 University of British Columbia, Department Of Pediatrics, Vancouver, Canada; 13 Claude Bernard University Lyon 1, Department Of Clinical Epidemiology, Lyon, France; 14 Université Claude Bernard Lyon 1, Faculty Of Medicine, Lyon, France; 15 Montreal Diet Dispensary, Dispensaire Diététique De Montréal, Montreal, Canada; 16 Maisonneuve-Rosemont Hospital, Department Of Anesthesiology And Pain Medicine,, Montreal, Canada; 17 Maisonneuve-Rosemont Hospital, Research Center, Montreal, Canada; 18 Zhengzhou University, College Of Public Health, Zhengzhou, China

**Keywords:** COVID-19 pandemic, maternal mental health during pregnancy, country/continent variations, Edinburgh Perinatal Depression Scale (EPDS)

## Abstract

**Introduction:**

Mental health regional differences during pregnancy through the COVID-19 pandemic is understudied.

**Objectives:**

We aimed to quantify the impact of the COVID-19 pandemic on maternal mental health during pregnancy.

**Methods:**

A cohort study with a web-based recruitment strategy and electronic data collection was initiated in 06/2020. Although Canadian women, >18 years were primarily targeted, pregnant women worldwide were eligible. The current analysis includes data on women enrolled 06/2020-11/2020. Self-reported data included mental health measures (Edinburgh Perinatal Depression Scale (EPDS), Generalized Anxiety Disorders (GAD-7)), stress. We compared maternal mental health stratifying on country/continents of residence, and identified determinants of mental health using multivariable regression models.

**Results:**

Of 2,109 pregnant women recruited, 1,932 were from Canada, 48 the United States (US), 73 Europe, 35 Africa, and 21 Asia/Oceania. Mean depressive symptom scores were lower in Canada (EPDS 8.2, SD 5.2) compared to the US (EPDS 10.5, SD 4.8) and Europe (EPDS 10.4, SD 6.5) (p<0.05), regardless of being infected or not. Maternal anxiety, stress, decreased income and access to health care due to the pandemic were increasing maternal depression. The prevalence of severe anxiety was similar across country/continents. Maternal depression, stress, and earlier recruitment during the pandemic (June/July) were associated with increased maternal anxiety.

**Conclusions:**

In this first international study on the impact of the COVID-19 pandemic, CONCEPTION has shown significant country/continent-specific variations in depressive symptoms during pregnancy, whereas severe anxiety was similar regardless of place of residence. Strategies are needed to reduce COVID-19’s mental health burden in pregnancy.

**Disclosure:**

No significant relationships.

